# Association of BRCA1- and BRCA2-deficiency with mutation burden, expression of PD-L1/PD-1, immune infiltrates, and T cell-inflamed signature in breast cancer

**DOI:** 10.1371/journal.pone.0215381

**Published:** 2019-04-25

**Authors:** Wei Xiong Wen, Chee-Onn Leong

**Affiliations:** 1 Center for Cancer and Stem Cell Research, International Medical University, Bukit Jalil, Kuala Lumpur, Malaysia; 2 School of Pharmacy, International Medical University, Bukit Jalil, Kuala Lumpur, Malaysia; University of Hawaii System, UNITED STATES

## Abstract

Immune checkpoint inhibitors have demonstrated effective anti-tumour response in cancer types with high mutation burden (e.g. melanoma) and in subset of cancers with features of genomic instability (e.g. mismatch-repair deficiency). One possible explanation for this effect is the increased expression of immune checkpoint molecules and pre-existing adaptive immune response in these cancers. Given that *BRCA1* and *BRCA2* are integral in maintaining genomic integrity, we hypothesise that the inactivation of these genes may give rise to breast cancers with such immunogenic phenotype. Therefore, using two large series of publicly available breast cancer datasets, namely that from The Cancer Genome Atlas and Wellcome Trust Institute, we sought to investigate the association between BRCA1- and BRCA2-deficiency with features of genomic instability, expression of *PD-L1* and *PD-1*, landscape of inferred tumour-infiltrating immune cells, and T-cell inflamed signature in breast cancers. Here, we report that BRCA1 and BRCA2-deficient breast cancers were associated with features of genomic instability including increased mutation burden. Interestingly, BRCA1-, but not BRCA2-, deficient breast cancers were associated with increased expression of *PD-L1* and *PD-1*, higher abundance of tumour-infiltrating immune cells, and enrichment of T cell-inflamed signature. The differences in immunophenotype between BRCA1- and BRCA2-deficient breast cancers can be attributed, in part, to *PTEN* gene mutation. Therefore, features of genomic instability such as that mediated by BRCA1- and BRCA2- deficiency in breast cancer were necessary, but not always sufficient, for yielding T cell-inflamed tumour microenvironment, and by extension, predicting clinical benefit from immunotherapy.

## Introduction

Immunotherapy using immune checkpoint blockade such as that of PD-1, PD-L1, and CTLA-4 inhibitors have demonstrated durable anti-tumour response in several cancer types including melanoma [1, 2], non-small cell lung carcinoma [3–5], head and neck squamous cell carcinoma [6], urothelial carcinoma [7], renal-cell carcinoma [8], and Hodgkin lymphoma [9]. Accordingly, selected immune checkpoint inhibitors have been approved by the US Food and Drug Administration (FDA) and European Medicine Agency (EMA) for the treatment of these cancers. Various predictors were found to be positively correlated with response to immune checkpoint inhibitors, in particular anti-PD-1 antibody, including high mutation burden and neoantigen load, increased expression of PD-L1, and increased expression of IFN-γ-responsive genes [7, 10–14]. Furthermore, biomarkers of genomic instability such as mismatch-repair deficiency and DNA repair pathway mutations including *POLE*, *POLD1*, and *MSH2* gave rise to similar genomic features and immunophenotype predictive of response to immunotherapy in several cancer types [12, 15, 16]. Collectively, these studies suggest that high mutation burden as a result of genomic instability and the consequent increased in tumour surface neoantigens leads to an increased in tumour-infiltrating immune cells and ultimately the compensatory up-regulation of the PD-1/PD-L1 pathway as a mechanism of inhibiting T-cell activation at tumour sites [17, 18].

On the other hand, immune checkpoint inhibitors in breast cancer demonstrated varying degrees of anti-tumour response depending breast cancer subtypes and the use of immune checkpoint inhibitors in monotherapy setting or in combination with chemotherapy or hormone therapy [19, 20]. Therefore, further research is required to identify breast cancer patients who are likely to benefit from immunotherapy.

Inherited mutations in *BRCA1* and *BRCA2* are associated with increased risk to breast cancer and are enriched in patients with an early age of diagnosis and family history of breast and ovarian cancer [21–23]. Somatic mutations in *BRCA1* and *BRCA2* mutations may also arise in sporadic cases of breast cancer [24, 25]. Inactivation of *BRCA1* and *BRCA2* via biallelic mutations and somatic hypermethylation (for *BRCA1*) gives rise to a mutational signature reflective of an underlying deficiency in homologous recombination repair [26]. Therefore, it is conceivable that genomic instability mediated by deficiency in BRCA1 and BRCA2 may give rise to immunophenotype in breast cancer predictive of response to immunotherapy such as increased expression of PD-L1 and PD-1 and higher abundance of tumour-infiltrating immune cells.

Here, using bioinformatics and statistical analysis on large series of publicly available breast cancer datasets [24, 25, 27], we formally evaluated the association between BRCA1- and BRCA2-deficiency with features of genomic instability, expression of *PD-L1* and *PD-1*, landscape of inferred tumour-infiltrating immune cells, and T cell-inflamed gene expression signature in breast cancers.

## Materials and methods

### Genomic and transcriptomic datasets

Two publicly available breast cancer datasets with both germline and somatic genomic sequencing data and transcriptomic sequencing data were used, namely samples from the Wellcome Sanger Institute (WSI) [25, 27] and The Cancer Genome Atlas [24].

For the WSI breast cancers, *BRCA1* and *BRCA2* germline and somatic mutation information, *BRCA1* and *BRCA2* copy number profile, and promoter hypermethylation status of *BRCA1* were retrieved from a previous report [27]. Catalogue of simple somatic mutations (point mutations and small indels) was downloaded from International Cancer Genome Consortium (ICGC) Data Portal (http://icgc.org/). Mutations were annotated as exonic or otherwise using ANNOVAR [28]. Gene expression values in log_2_(FPKM) and clinical and pathology information were similarly available from an earlier report [25]. Gene expression values were transformed as *X'* = log_2_(*X* + 1) where *X* represents the normalized fragments per kilobase transcript per million mapped reads values (FPKM).

For TCGA breast cancers, *BRCA1* and *BRCA2* germline and somatic mutation information was similarly retrieved from a recent study [29] whereas *BRCA1* and *BRCA2* copy number profiles were downloaded from Broad GDAC FIREHOSE (https://gdac.broadinstitute.org/) and *BRCA1* HK27 methylation data values were downloaded from cBioPortal for Cancer Genomics (http://www.cbioportal.org/). TCGA breast cancers with *BRCA1* and *BRCA2* homozygous deletion and *BRCA1* promoter hypermethylation were subsequently determined as previously described [30]. Catalogue of simple somatic mutations (point mutations and small indels) and RSEM-normalized gene expression values were downloaded from Broad GDAC FIREHOSE. Gene expression values were transformed as *X'* = log_2_(*X* + 1) where *X* represents the normalized FPKM. Duplicate samples in TCGA were remove using the collapseRows function from the R package *WGCNA* by selecting the samples with the maximum mean expression value [31].

Breast cancer samples with germline and somatic biallelic inactivation and homozygous deletion of *BRCA1* and *BRCA2*, and promoter hypermethylation of *BRCA1* were classified as BRCA1- or BRCA2-deficient breast cancers as previously described [26, 27]. All other samples were classified as BRCA-proficient breast cancers. For the genomic analysis, 560 breast cancers (483 BRCA-proficient, 47 BRCA1-deficient, and 30 BRCA2-deficient) from WSI and 858 breast cancers (804 BRCA-proficient, 31 BRCA1-deficient, and 23 BRCA2-deficient) from TCGA with BRCA1 and BRCA2 status and mutation data were included in this study. For the transcriptomic analysis, 266 breast cancers (224 BRCA-proficient, 27 BRCA1-deficient, and 15 BRCA2-deficient) from WSI and 927 breast cancers (872 BRCA-proficient, 31 BRCA1-deficient, and 24 BRCA2-deficient) from TCGA with BRCA1 and BRCA2 status and gene expression profile were included in this study.

### Mutational signature and neoantigen analysis

The deconstructSigs program was used to determine the proportion of known mutational signatures from somatic single base substitutions [32]. Predicted neoantigen data available for TCGA breast cancers were collated from two previous reports [33, 34].

### Identification of immune infiltrate subpopulations and T cell-inflamed signature

Twenty-eight immune cell types were characterized with TIminer from tumour gene expression profiles [35]. T cell-inflamed signature for each sample were calculated by finding the mean expression of 18 IFN-γ and T cell-associated inflammatory genes shown to be predictive of response to the PD-1 immune checkpoint inhibitor across different tumour types [10].

### Breast cancer subtype classification

PAM50 subtype classifier described by Parker *et al*. was used to classify tumours into five intrinsic molecular subtypes using the R package *genefu* [36]. Triple negative breast cancers were defined as estrogen receptor (ER) negative, progesterone receptor (PR) negative, and human epidermal growth factor 2 (HER2) receptor negative. In TCGA, HER2 immunohistochemistry score of 3+ was considered as positive while scores of 0, 1+, and 2+ were considered as negative.

### Clinicopathological features

Patient characteristics including age of diagnosis, menopausal status, tumour grade, and hormone receptor status were collated for both WSI and TCGA. For breast cancers from WSI, patient characteristics were retrieved from a previous report [25]. For breast cancers from TCGA, all patient characteristics except tumour grade were downloaded from Broad GDAC FIREHOSE whereas tumour grade was retrieved from a previous publication [37].

### *PTEN* mutation and PTEN protein expression analysis

Phosphate and tensin homolog (PTEN) protein expression of TCGA breast cancer samples derived from reverse phase protein array (RPPA) was downloaded from GDAC. *PTEN* copy number alteration (CNA) and point mutation status were downloaded from cBioPortal. In total, 662 samples with *PTEN* CNA and point mutation status, PTEN protein expression, BRCA1- and BRCA2-deficient status, and T cell-inflamed signature score available were included in this analysis.

### Statistical analysis

Wilcoxon rank sum test was used to estimate the significance of association between a continuous variable and a binary variable. Fisher exact test of independence was used to estimate the significance of association between two categorical variables. Correlation between BRCA1 and BRCA2 status, clinicopathological features, and PAM50 molecular subtype with T cell-inflamed signature was estimated using linear regression model. Statistical tests were considered significant based on two-sided hypothesis tests with *P* < 0.05. All statistical analysis was performed in the R programming environment (version 3.4.3).

## Results

### Characteristics of breast cancer patients from WSI and TCGA

Breast cancer patients from WSI had significantly younger age of diagnosis, higher proportion of pre-menopausal patients, higher proportion of high-grade tumours, and higher proportion of ER- and PR-negative, and triple-negative breast cancers compared to that of TCGA (S1 Table). The proportion of BRCA1- and BRCA2-deficient breast cancers was also significantly higher in WSI compared to TCGA. This may be attributed to the younger age of diagnosis as well as the higher proportion of pre-menopausal patients in WSI compared to TCGA as the proportion of patients who are carrier of breast cancer predisposition genes, such as *BRCA1* and *BRCA2*, are anticipated to be higher in younger populations of breast cancer patients [22, 23]. Indeed, the proportion of BRCA1- and BRCA2-deficient breast cancers with biallelic pathogenic germline mutations was significantly higher in WSI compared to TCGA whereas the proportion of BRCA1- and BRCA2-deficient breast cancers with biallelic pathogenic somatic mutations was not significantly different between the two cohorts.

### Homologous recombination repair deficient mutational signature and chromosomal instability in BRCA1- and BRCA2-deficient breast cancers

**Fig 1:**
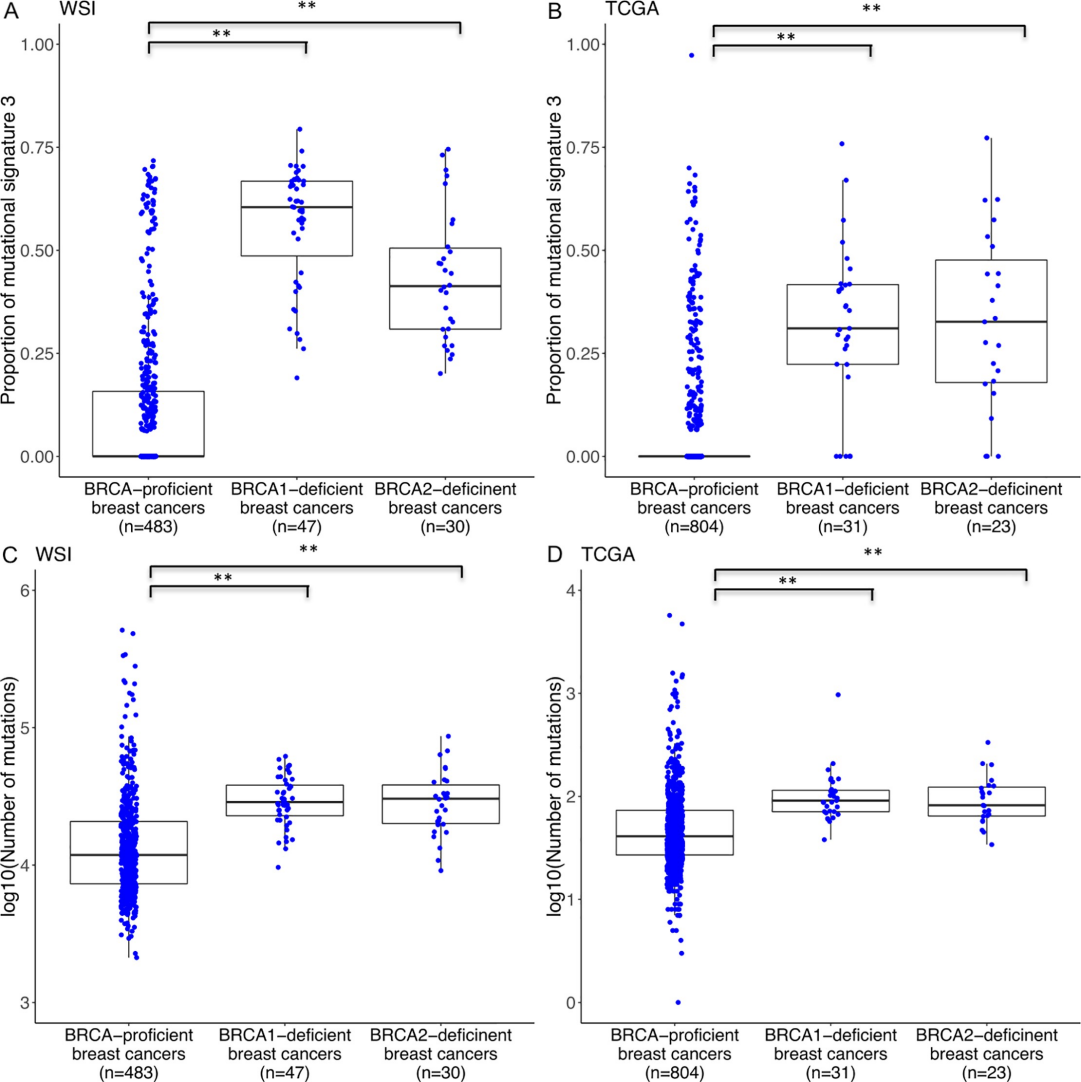
Proportion of mutational signature 3 and overall mutation burden by BRCA status in WSI and TCGA breast cancers. ** *P* < 0.01.

BRCA1- and BRCA2-deficient breast cancers from WSI and TCGA had significantly higher proportion of homologous recombination repair deficient mutational signature (signature 3 as per nomenclature by Alexandrov *et al*. [38]). BRCA1- and BRCA2-deficient breast cancers from WSI had 0.60 and 0.41 median proportion of signature 3 compared to median of 0 in BRCA-proficient breast cancers (*P* = 2.2 x 10^−28^ and 1.9 x 10^−16^, respectively; Fig 1A). Similarly, BRCA1- and BRCA2-deficient breast cancers from TCGA had 0.31 and 0.33 median proportion of signature 3 compared to median of 0 in BRCA-proficient breast cancers (*P* = 3.5 x 10^−20^ and 3.4 x 10^−18^, respectively; Fig 1B). The proportion of signature 3 remained associated with BRCA1- and BRCA2-deficiency in the whole-genome sequenced breast cancers from WSI when only exonic mutations were analysed with median proportion of 0.39 and 0.20, respectively, compared to median of 0 in BRCA-proficient breast cancers (*P* = 3.2 x ^10–28^ and 3.6 x 10^−9^, respectively; S1 Fig). Further profiling of the ‘CIN25’ signature [39], a gene expression signature for chromosomal instability, in breast cancers from WSI and TCGA showed that BRCA1- and BRCA2-deficient breast cancers generally overexpressed this gene set compared to BRCA-proficient breast cancers (S2 Fig).

### Overall mutation burden and neoantigen load in BRCA1- and BRCA2-deficienct breast cancers

BRCA1- and BRCA2-deficient breast cancers had significantly higher number of reported mutations with the median number of mutations in WSI BRCA1- and BRCA2-deficient breast cancers being 2.4 and 2.6 fold higher, respectively, compared to BRCA-proficient breast cancers (*P* = 1.1 x 10^−12^ and 6.4 x 10^−8^, respectively; Fig 1C). Similarly, the median number of mutations in TCGA BRCA1- and BRCA2-deficient breast cancers being 2.2 and 2.0 fold higher, respectively, compared to BRCA-proficient breast cancers (*P* = 6.6 x 10^−9^ and 6.0 x 10^−6^, respectively; Fig 1D). When only exonic mutations were analysed in the whole-genome sequenced breast cancers from WSI, BRCA1- and BRCA2-deficient breast cancers remained associated with higher mutation burden with medians being 2.2 and 2.1 fold higher, respectively, compared to BRCA-proficient breast cancers (*P* = 3.5 x 10^−11^ and 7.4 x 10^−6^, respectively; S3 Fig). Furthermore, using neoantigen data reported by Rooney *et al*. [33], the median number of predicted neoantigens in TCGA BRCA1- and BRCA2-deficient breast cancers were observed to be 2.3 and 2.5 fold higher, respectively, compared to BRCA-proficient breast cancers (*P* = 5.4 x 10^−7^ and 3.3 x 10^−6^, respectively; S4 Fig). Similarly, using neoantigen data reported by Charoentong *et al*. [34], the median number of predicted neoantigens in TCGA BRCA1- and BRCA2-deficient breast cancers were found to be 2.0 and 2.3 fold higher, respectively, compared to BRCA-proficient breast cancers (*P* = 2.0 x 10^−6^ and 2.0 x 10^−4^, respectively).

### Expression of *PD-L1* and *PD-1* in BRCA1- and BRCA2-deficient breast cancers

**Fig 2:**
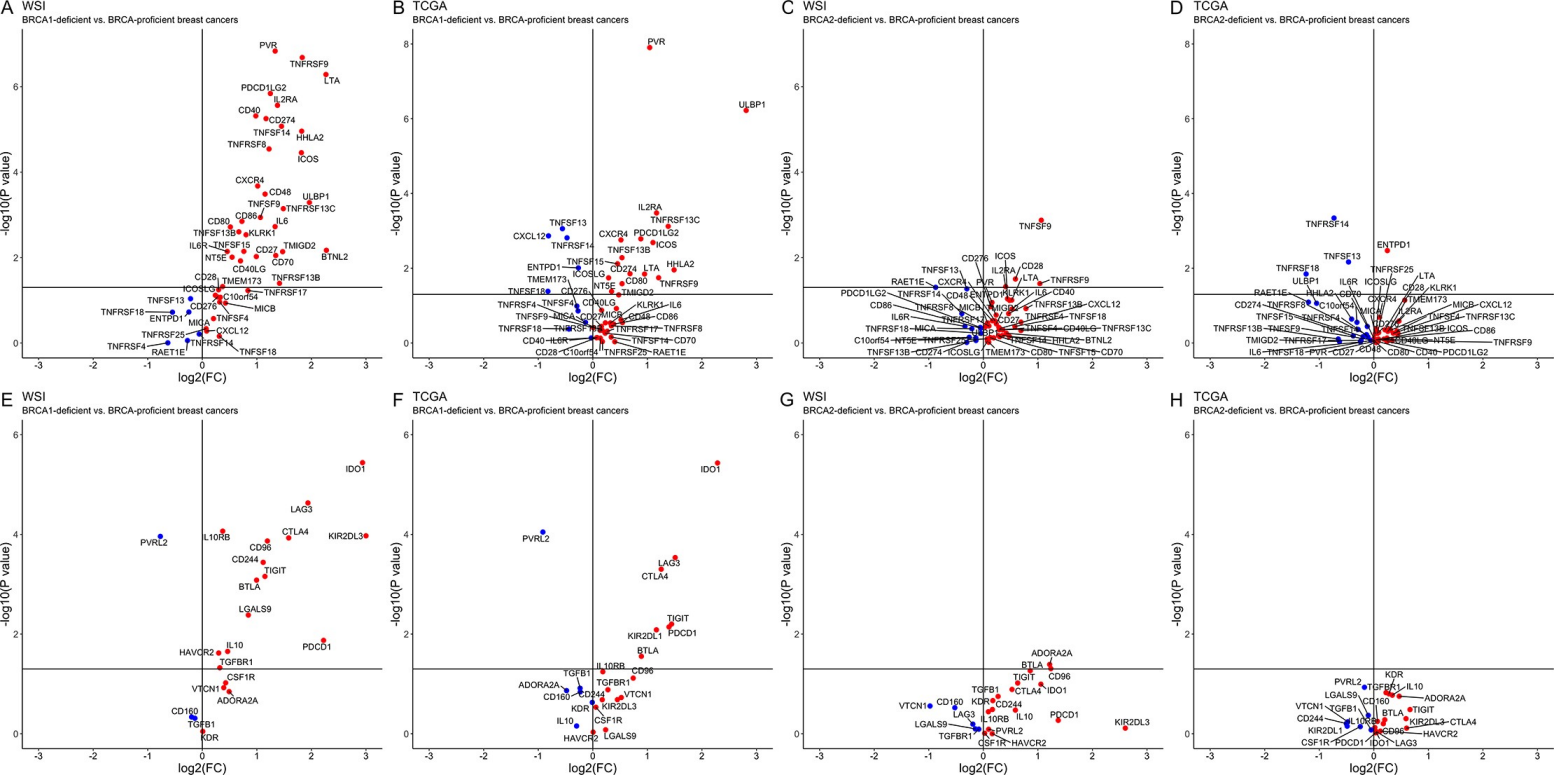
**Volcano plots of immunostimulator (A-D) and immunoinhibitor (E-H) molecules in BRCA1- and BRCA2-deficient vs. BRCA-proficient breast cancers from WSI and TCGA.** FC: fold change. *KIR2DL3* with log2FC 14.5 in BRCA1-deficient vs. BRCA-proficient breast cancers from WSI shown at the boundary of the plot.

The features genomic instability observed in both BRCA1- and BRCA2-deficient breast cancers suggests that these cancers would demonstrate similar gene expression signatures and immune features. Interestingly, BRCA1-, but not BRCA2-, deficient breast cancer was associated with higher *PD-L1* (*CD274*) expression compared to BRCA-proficient breast cancers in both WSI and TCGA cohorts (*P*_BRCA1_ = 5.6 x 10^−6^ and 0.014, respectively. *P*_BRCA2_: 0.97 and 0.67, respectively; Fig 2A–2D). Fold changes in log2 of *PD-L1* expression for BRCA1-deficient breast cancers compared BRCA-proficient breast cancers from WSI and TCGA were 1.2 and 0.68, respectively. Similarly, BRCA1-, but not BRCA2-, breast cancers was also associated with higher *PD-1* (*PDCD1*) expression compared to BRCA-proficient breast cancers in both WSI and TCGA cohorts (*P*_BRCA1_: 0.013 and 7.2 x 10^−3^, respectively. *P*_BRCA2_: 0.54 and 0.96, respectively; Fig 2E–2H). Fold changes in log2 of *PD-1* expression for BRCA1-deficient breast cancers compared BRCA-proficient breast cancers from WSI and TCGA were 2.2 and 1.4, respectively. Expression of immunostimulator and immunoinhibitor molecules [34], other than *PD-L1* and *PD-1*, respectively, were generally increased in BRCA1-deficient breast cancers, but not in BRCA2-deficient breast cancers, compared to BRCA-proficient breast cancers. Notably, *PVR* was the most significantly up-regulated immunostimulator molecule in BRCA1-deficient breast cancers compared to BRCA-proficient breast cancers whereas *IDO1* and *LAG3* were the top most significantly up-regulated immunoinhibitor molecules in BRCA1-deficient breast cancers compared to BRCA-proficient breast cancers in both WSI and TCGA.

### Pattern of immune infiltrates in BRCA1- and BRCA2-deficient breast cancers

**Fig 3:**
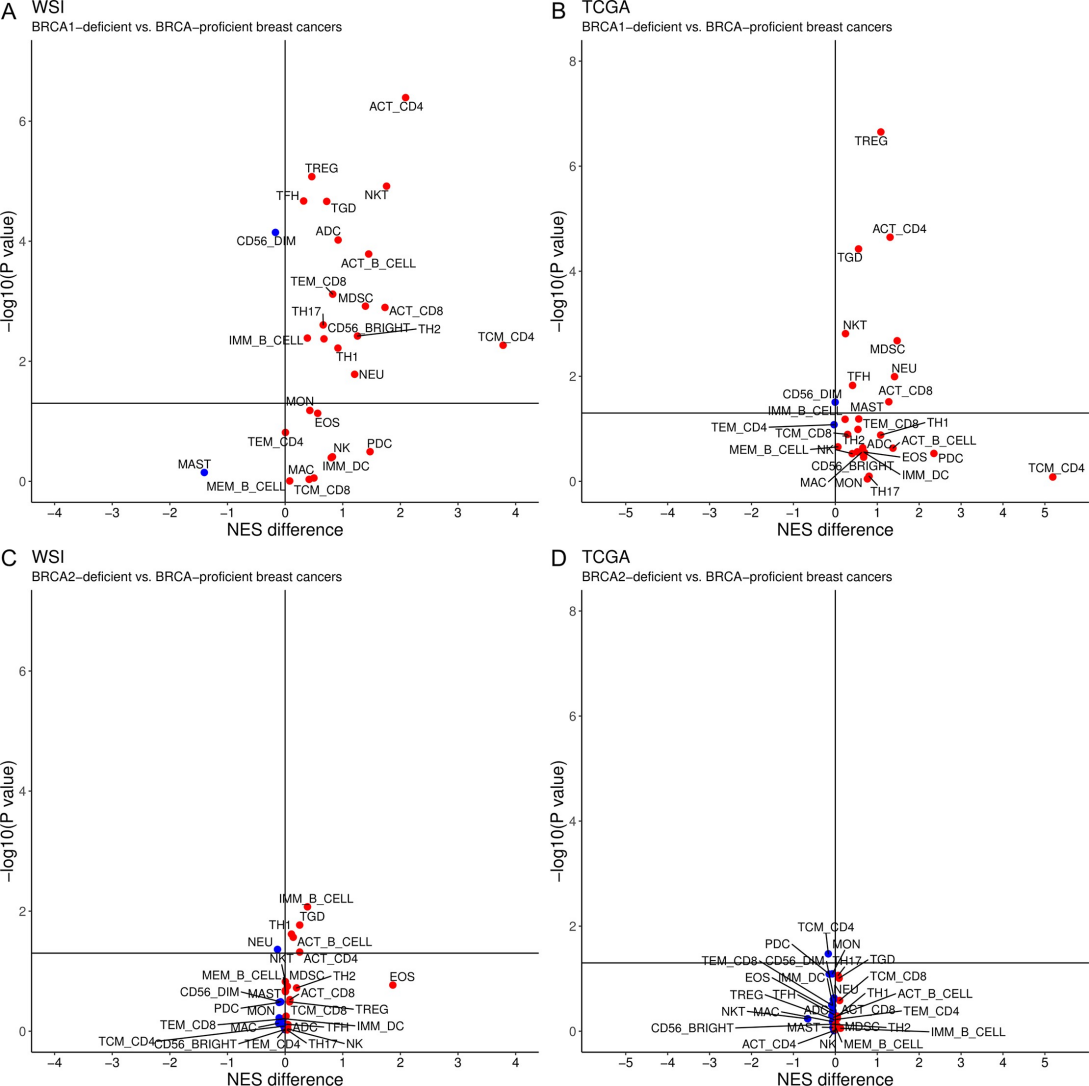
**Relative enrichment of immune infiltrates in BRCA1- (A-B) and BRCA2-deficient (C-D) breast cancers compared to BRCA-proficient breast cancers from WSI and TCGA.** ACT_B_cell: Activated B cell, ACT_CD4: Activated CD4 T cell, ACT_CD8: Activated CD8 T cell, ADC: Activated dendritic cell, CD56_BRIGHT: CD56bright natural killer cell, CD56_DIM: CD56dim natural killer cell, EOS: Eosinophil, IMM_B_CELL: Immature B cell, IMM_DC: Immature dendritic cell, MAC: Macrophage, MAST: Mast cell, MDSC: Myeloid-derived suppressor cell, MEM_B_CELL: Memory B cell, MON: Monocyte, NEU: Neutrophil, NK: Natural killer cell; NES: Normalized enrichment score, NKT: Natural killer T cell, PDC: Plasmacytoid dendritic cell, TCM_CD4: Central memory CD4 T cell, TCM_CD8: Central memory CD8 T cell, TEM_CD4: Effector memory CD4 T cell, TEM_CD8: Effector memory CD8 T cell, TFH: T follicular helper cell, TGD: γδ T cell, TH1: Type 1 T helper cell, TH17: Type 17 helper cell, TH2: Type 2 T helper cell, TREG: Regulatory T cell.

As combinations of immunomodulatory molecules, including PD-L1 and PD-1, are expressed by tumour and tumour-infiltrating immune cells to shape the landscape of the tumour microenvironment, we investigated whether the differences in expression of immunomodulatory molecules observed between BRCA1- and BRCA2-deficent breast cancers correspond to distinct patterns of immune filtrates. Immune cell subtypes that were enriched in BRCA1-deficient breast cancers relative to BRCA-proficient breast cancers in WSI and TCGA include cells involved in the adaptive immune response (activated CD4 T cells, activated CD8 T cells, regulatory T cells, T follicular helper cells, and γδ T cells) and innate immunity (myeloid-derived suppressor cell and natural killer cells) while CD56^dim^ natural killer cells were depleted in BRCA1-deficient breast cancers relative to BRCA-proficient breast cancers (Fig 3A and 3B). BRCA2-deficient breast cancers in WSI were enriched for activated CD4 T cells, γδ T cells, type 1 T helper cell, activated B cells, and immature B cell compared to BRCA-proficient breast cancers. However, none of the immune cell subtypes were observed to be significantly enriched in BRCA2-deficient breast cancers compared to BRCA-proficient breast cancers in TCGA (Fig 3C and 3D). Collectively, these demonstrated that BRCA1- and BRCA2-deficient breast cancers have different immunophenotype notwithstanding the similar genomic features shared between these groups.

### Association between BRCA1- and BRCA2-deficient breast cancers and clinicopathological features with T cell-inflamed signature

A T cell-inflamed gene expression profile consisting of 18 genes was determined previously to be associated with response to immune checkpoint inhibitors, specifically PD-1 inhibitor [10]. Here, we investigated the relationship between BRCA1- and BRCA2-deficiency with T cell-inflamed signature. As expected based on *PD-L1* and *PD-1* expression and immune infiltrate profile, BRCA1-, but not BRCA2-, deficient breast cancers were associated with higher T cell-inflamed signature in WSI (*P* = 1.9 x 10^−7^ and 0.072, respectively) and TCGA (*P* = 8.0 x 10^−3^ and 0.95, respectively) (Table 1). Clinicopathological features associated with increased T cell-inflamed signature in WSI and TCGA included young age of diagnosis, tumour grade III, ER- and PR-negative breast cancers, triple-negative breast cancers, and basal-like subtype. In TCGA, HER2-positive breast cancers and HER2-enriched subtype were associated with higher T cell-inflamed signature.

**Table 1:** Univariate linear regression analysis of BRCA1/2 status, clinicopathological features, and PAM50 subtypes with T cell-inflamed signature score.

Characteristic	WSI		TCGA	
	β (S.E.)	*P* value	β (S.E.)	*P* value
BRCA status				
BRCA1-deficient ^a^	0.91 (0.17)	1.9 x 10^−7^	0.72 (0.27)	8.0 x 10^−3^
BRCA2-deficient ^a^	0.40 (0.22)	0.072	0.02 (0.30)	0.95
Age (≤ median) ^b^	0.31 (0.11)	5.8 x 10^−3^	0.30 (0.10)	2.0 x 10^−3^
Grade ^c^				
II	0.22 (0.20)	0.28	0.29 (0.22)	0.19
III	0.54 (0.20)	6.0 x 10^−3^	0.95 (0.22)	1.9 x 10^−5^
ER-negative ^d^	0.80 (0.11)	2.8 x 10^−12^	0.84 (0.11)	5.6 x 10^−13^
PR-negative ^d^	0.64 (0.11)	8.3 x 10^−9^	0.56 (0.10)	8.4 x 10^−8^
HER2-negative ^d^	0.09 (0.44)	0.83	-0.39 (0.17)	0.023
TNBC ^e^	0.81 (0.11)	4.5 x 10^−12^	0.83 (0.16)	7.4 x 10^−7^
PAM50 ^f^				
Luminal B	0.13 (0.11)	0.25	-0.14 (0.12)	0.21
Basal-like	1.06 (0.12)	1.6 x 10^−15^	0.87 (0.14)	4.1 x 10^−10^
HER2-enriched	0.29 (0.24)	0.23	0.82 (0.16)	4.1 x 10^−7^
Normal-like	0.12 (0.78)	0.87	0.02 (0.20)	0.93

^a^ BRCA-proficient breast cancers as reference

^b^ Age (> median) as reference

^c^ Grade I as reference

^d^ Receptor positive as reference

^e^ Non-TNBC as reference

^f^ Luminal A as reference

Incidentally, young age of diagnosis, high-grade tumours, triple-negative breast cancers, and basal-like subtype are known to be associated with BRCA1-deficient breast cancers [22, 23]. Similarly, these features were found to be enriched in BRCA1-deficient breast cancers from WSI and TCGA (S2 Table). To determine whether the relationship between BRCA1-deficiency and T cell-inflamed signature was related to these features, we adjusted for these features in a multiple linear regression model. The association between BRCA1-deficiency and T cell-inflamed signature was attenuated in this model (S3 Table), suggesting that these features may have contributed to this phenomenon.

### Immunophenotype of BRCA1- and BRCA2-deficient breast cancers is related to *PTEN* mutation

**Fig 4:**
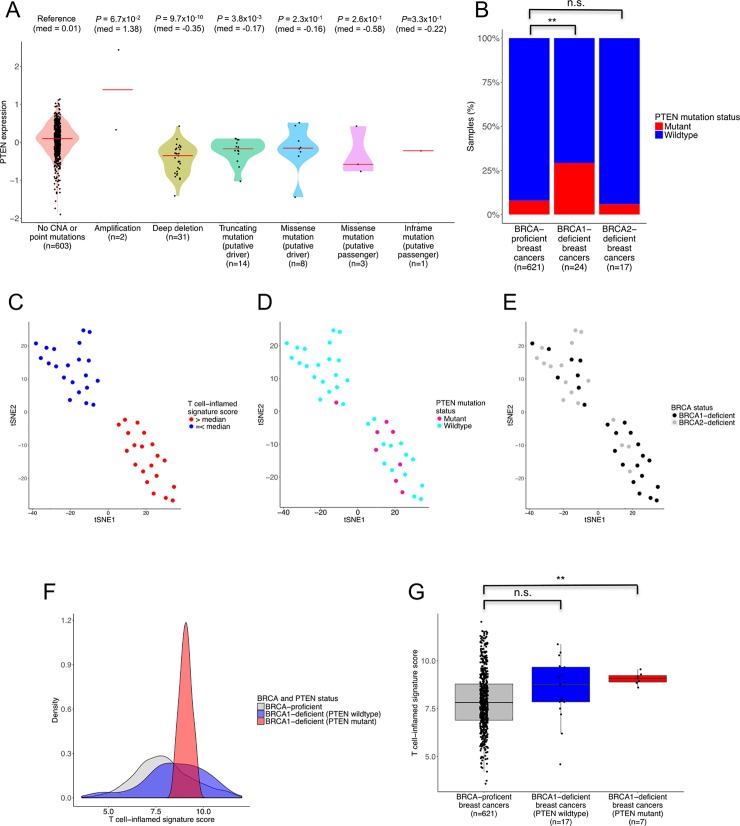
Association between BRCA status and T cell-inflamed signature in relation to *PTEN* mutation status. **(A)** PTEN protein expression stratified by *PTEN* copy number alteration and point mutation. **(B)** Proportion of *PTEN* mutant samples stratified by BRCA status. **(C-E)** tSNE visualisation of BRCA1- and BRCA2-deficient breast cancers. Colour represents T cell-inflamed signature score divided at the median, *PTEN* mutation status, and BRCA status, respectively. **(F-G)** Distribution of T cell-inflamed signature scores in BRCA-proficient and BRCA1-deficient breast cancers with and without *PTEN* mutation. n.s.: Not statistically significant, ** *P* < 0.01.

While both BRCA1- and BRCA2-deficient breast cancers shared similar genomic features, only BRCA1-, but not BRCA2-deficient breast cancers, were associated with immune signatures. PTEN loss has been shown to up-regulate PD-L1 expression via PI3K pathway in several solid cancers [40–42]. Moreover, PTEN loss has also been shown to be enriched in *BRCA1* mutation carriers [43, 44]. Therefore, we hypothesized that the difference in immunophenotype between BRCA1- and BRCA2-deficient breast cancers may be attributed to PTEN loss, such as that mediated by inactivating *PTEN* gene mutations. We retrieved reverse phase protein array (RPPA) data for TCGA to determine the link between BRCA-deficiency and T cell-inflamed signature in relation to PTEN loss due to *PTEN* gene mutation. We observed *PTEN* copy number loss and point mutation to be associated with lower levels of PTEN protein expression whereas *PTEN* copy number gain was associated with higher levels of PTEN protein expression relative to samples without and reported *PTEN* gene aberrations (Fig 4A). We subsequently classified samples with *PTEN* copy number loss or point mutation as *PTEN* mutant breast cancers, and samples with *PTEN* copy number gain or without any reported *PTEN* copy number alterations or point mutations as *PTEN* wildtype breast cancers. The proportion of *PTEN* mutant samples in BRCA-proficient, and in BRCA1- and BRCA2-deficient breast cancers was 7.9%, 29%, and 5.9%, respectively (Fig 4B). *PTEN* mutant samples were significantly enriched in BRCA1-, but not BRCA2-, deficient breast cancers compared to BRCA-proficient breast cancers (*P* = 2.8 x 10^−3^ and >0.99, respectively).

T-distributed stochastic neighbour embedding (tSNE) analysis using the individual genes that constituted the T cell-inflamed signature score revealed two distinct clusters of BRCA1/2-deficient breast cancers (Fig 4C). The first group consist of BRCA1/2-deficient breast cancers with T cell-inflamed signature score above the median (T^high^) while the second group consist of BRCA1/2-deficient breast cancers with T cell-inflamed signature score equal to or below the median (T^low^). All but one sample with *PTEN* mutation clustered within the T^high^ group (Fig 4D). Notably, all BRCA1-deficient breast cancers with *PTEN* mutation clustered within the T^high^ group whereas the sole BRCA2-deficient breast cancer with *PTEN* mutation clustered within the T^low^ group (Fig 4E).

BRCA1-deficient breast cancers with *PTEN* mutation generally had higher T cell-inflamed signature scores compared to BRCA1-proficient breast cancers and BRCA1-deficient breast cancers without *PTEN* mutation (Fig 4F). Notably, BRCA1-deficient breast cancers with *PTEN* mutation, but not BRCA1-deficient breast cancers without *PTEN* mutation, had significantly higher T cell-inflamed signature scores compared to BRCA-proficient breast cancers (*P* = 9.3 x 10^−3^ and 0.07, respectively; Fig 4G). Collectively, these data suggest that the differences in immunophenotype between BRCA1- and BRCA2-deficient breast cancers may be attributed, in part, to *PTEN* gene mutation.

## Discussion

In this study, we investigated the features of genomic instability, expression of checkpoint molecules, and T cell-inflamed signature stratified by BRCA1- and BRCA2-deficiency status in two large series of breast cancer patients.

BRCA1- and BRCA2-deficient breast cancers were shown to be associated with elevated proportion of signature 3, suggesting an underlying deficiency in homologous recombination repair, and by extension, higher degree of genomic instability in these groups of breast cancers. Indeed, BRCA1- and BRCA2-deficient breast cancers were found to be enriched for CIN25 signature that is a biomarker for chromosomal instability [39]. Nevertheless, the presence of breast cancers within the BRCA-proficient group that demonstrated signature 3 activity suggests that there may be samples in which have hitherto defects not identified in *BRCA1* or *BRCA2* or defects in other homologous recombination repair genes such as that of *PALB2* and *RAD51C* [26]. BRCA1- and BRCA2-deficient breast cancers were further shown to have higher number of mutations and correspondingly and higher number of predicted neoantigens. Taken together, BRCA1- and BRCA2-deficient breast cancers demonstrated higher degree of genomic instability compared to BRCA-proficient breast cancers. Therefore, based on previous studies that reported an association between genomic instability and immunophenotype characterised by increased expression of checkpoint inhibitor molecules and infiltration of immune cells primarily involved in adaptive immune response [15, 45, 46], it was conceivable that both BRCA1- and BRCA2-deficient breast cancers may display such immunophenotype.

Interestingly, BRCA1-, but not BRCA2-, deficient breast cancers were observed to be associated with higher expression of *PD-L1* and *PD-1*, and increased abundance of tumour-infiltrating immune cells involved in adaptive immune response. To the best of our knowledge, the expression of *PD-L1* and *PD-1* and the abundance of a comprehensive panel of tumour-infiltrating immune cells in BRCA1- and BRCA2-deficient breast cancers have hitherto not been described. It is noteworthy that an on-going clinical trial is investigating the association of inherited mutations in DNA repair genes, including *BRCA1* and *BRCA2*, with PD-L1 expression and immune cells in breast cancer patients (ClinicalTrials.gov identifier: NCT03495544).

A T cell-inflamed gene expression profile was recently shown to be predictive of response to immunotherapy, specifically anti-PD-1 therapies. Herein, we investigated the relationship between BRCA1- and BRCA2-deficiency and clinicopathological features with T cell-inflamed signature. As expected, BRCA1-, but not BRCA2-, deficient breast cancers were associated with increased T cell-inflamed signature. Interestingly, clinicopathological features associated with BRCA1-deficiency such as younger age at diagnosis, high-grade tumour, triple-negative breast cancer and basal-like subtype [22, 23] were similarly associated with increased T cell-inflamed signature. Accordingly, we showed in a multiple linear regression model that the enrichment of T cell-inflamed signature in BRCA1-deficient breast cancers can be attributed, at least in part, to these clinical features.

BRCA2-deficient breast cancers displayed similar features of genomic instability as that in BRCA1-deficient breast cancers but did not demonstrate similar immunophenotype as that of BRCA1-deficient breast cancers. Loss of PTEN has been shown to increase PD-L1 expression via the PI3K pathway in several cancer types including triple-negative breast cancer [40–42]. Furthermore, PTEN loss was reported to be enriched in *BRCA1*-mutated breast cancers [43, 44]. Therefore, it is conceivable that PTEN loss, such as that mediated by inactivating *PTEN* gene mutation, may be one mechanism underlying the differences in immunophenotype between BRCA1- and BRCA2-deficient breast cancers. Indeed, we shown that BRCA1-deficient breast cancers with *PTEN* mutation, but not BRCA1-deficient breast cancers without *PTEN* mutation, to be associated with T cell-inflamed signature. Nevertheless, the paucity of BRCA2-deficient breast cancers with *PTEN* mutation in this cohort (n = 1) precluded comparison of T cell-inflamed signature with BRCA2-deficient breast cancers without *PTEN* mutation. These data suggest that PTEN loss, mediated by *PTEN* gene mutation, may be one possible mechanism by which BRCA1- and BRCA2-deficient breast cancers give rise to different immunophenotype.

In a recent study, antitumour immune response was demonstrated in *Brca1*-mutated mice when treated with cisplastin with combined dual anti-PD-1 and anti-CTLA4 therapy. However, antitumour immune response to similar treatment was not investigated in *Brca2*-mutated mice [47]. Future work may be warranted to investigate additional mechanism by which BRCA1- and BRCA2-deficient breast cancers give rise to different immunophenotype. It is noteworthy that an on-going clinical trial is investigating pembrolizumab, an anti-PD-1 antibody, in advanced *BRCA1*- and *BRCA2*-mutated breast cancer patients (ClinicalTrials.gov identifier: NCT03025035).

BRCA1- and BRCA2-deficient high-grade ovarian cancers (HGSOCs) were shown to have increased neoantigen burden, increased immune infiltrates, and higher PD-L1 and PD-1 expression compared to BRCA-proficient ovarian cancers [48]. BRCA1- and BRCA2-deficient ovarian cancers in an unselected cohort of ovarian cancer patients were also shown to have increased PD-L1 and PD-1 expression compared to BRCA-proficient ovarian cancers. Interestingly, high-grade ovarian cancers were also shown to have increased *PD-L1* and *PD-1* expression in this study [49]. In light of the evidence provided by our study, it may be of particular interest to investigate the impact of BRCA1- and BRCA2-deficiency, separately, on the PD-L1 and PD-1 expression and the immune landscape in ovarian cancers as these studies considered BRCA1- and BRCA2-deficient ovarian cancers as a single entity [48, 49]. Notably, a recent study identified *BRCA1*, but not *BRCA2*, mutations to be associated with immunogenic phenotype in ovarian cancers [50].

Taken together, our analysis suggest that genomic instability and the consequent increased in mutation burden in breast cancers mediated by BRCA1- and BRCA2-deficiency may not be the sole determinant of *PD-L1* and *PD-1* expression, immune infiltrates, and T cell-inflamed signature, and therefore may be insufficient to predict clinical benefit from immunotherapy.

The limitation of our study was the small number of BRCA1- and BRCA2-deficient breast cancers relative to BRCA-proficient breast cancers. The proportion of BRCA1- and BRCA2-deficient breast cancers in WSI available for genomic and transcriptomic analysis was 14% and 16%, respectively, whereas the proportion of BRCA1- and BRCA2-deficient breast cancers in TCGA available for genomic and transcriptomic analysis was 6.3% and 5.9%, respectively. Therefore, further validation in study populations consisting of higher proportion of BRCA1- and BRCA2-deficient breast cancers, such as that in breast cancer cohorts enriched with *a prior* high-risk patients, may be warranted to assess the generalizability of our findings.

## Supporting information

S1 Fig Proportion of mutational signature 3 by BRCA status in WSI when only exonic mutations were analysed. ** *P* < 0.01.
(TIFF)

S2 Fig ‘CIN25’ signature in WIS and TCGA breast cancers by BRCA status. (TIFF)

S3 Fig Overall mutation burden by BRCA status in WSI breast cancers when only exonic mutations were analysed. ** *P* < 0.01.
(TIFF)

S4 Fig Neoantigen load in TCGA breast cancers based on neoantigen data from Rooney et al. and Charoentong et al.. ** *P* < 0.01.
(TIFF)

S1 Table Clinicopathological characteristics and proportion of BRCA1/2 mutation carriers of breast cancer patients from WSI and TCGA. (PDF)

S2 Table Clinicopathological characteristics and PAM50 subtypes of BRCA1/2-deficient vs. BRCA-proficient breast cancers in WSI and TCGA. (PDF)

S3 Table Multiple linear regression of BRCA1/2 status and T-cell inflamed signature score adjusted for clinicopathological features and PAM50 subtypes. (PDF)
