# Supplementary figures and images for: Association of BRCA1- and BRCA2-deficiency with mutation burden, expression of PD-L1/PD-1, immune infiltrates, and T cell-inflamed signature in breast cancer
Source: PLoS One. 2019 Apr 25;14(4):e0215381. doi: 10.1371/journal.pone.0215381 (PMC6483182; doi:10.1371/journal.pone.0215381)

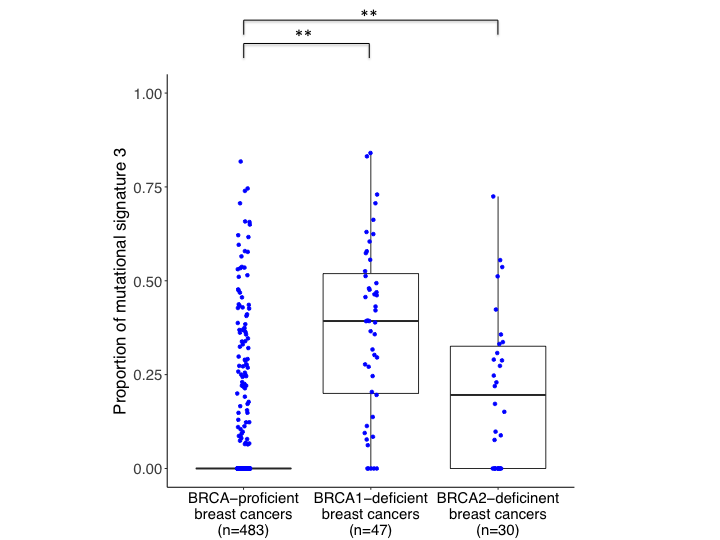

Supplement: S1 Fig — ** P < 0.01. (TIFF) [file pone.0215381.s001.tiff]

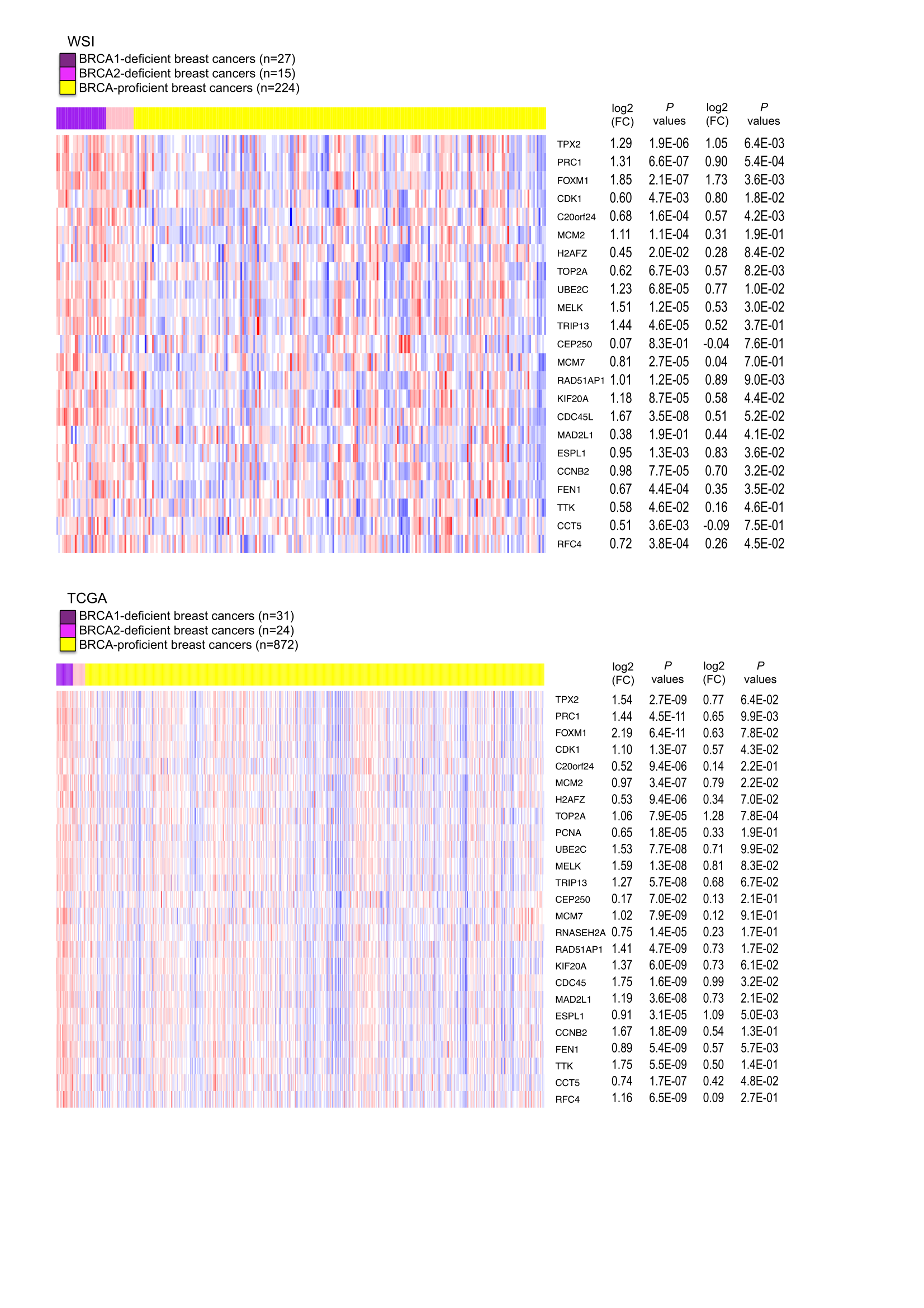

Supplement: S2 Fig — (TIFF) [file pone.0215381.s002.tiff]

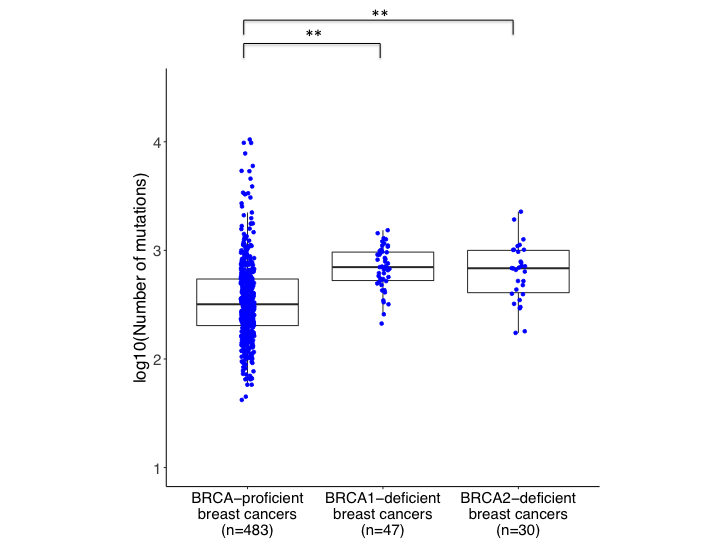

Supplement: S3 Fig — ** P < 0.01. (TIFF) [file pone.0215381.s003.tiff]

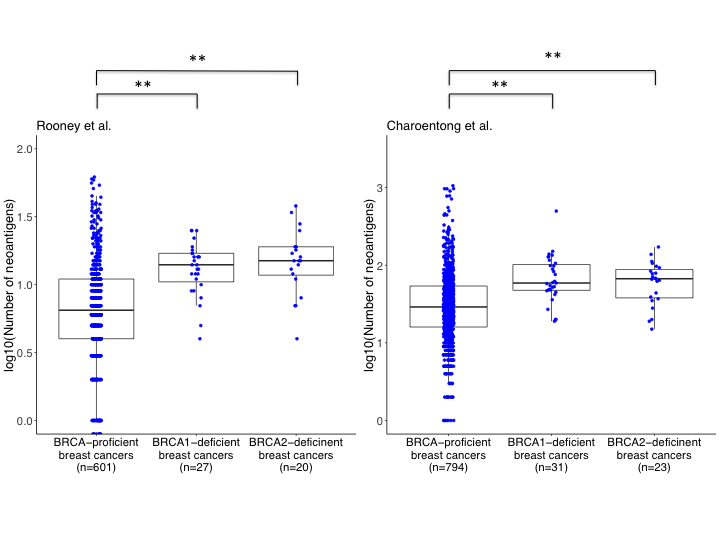

Supplement: S4 Fig — ** P < 0.01. (TIFF) [file pone.0215381.s004.tiff]
