# Supplementary material for: Association of BRCA1- and BRCA2-deficiency with mutation burden, expression of PD-L1/PD-1, immune infiltrates, and T cell-inflamed signature in breast cancer
Source: PLoS One. 2019 Apr 25;14(4):e0215381. doi: 10.1371/journal.pone.0215381 (PMC6483182; doi:10.1371/journal.pone.0215381)
Supplement: S1 Table — (PDF) [file pone.0215381.s005.pdf]

Supplementary table 1: Clinicopathological characteristics and proportion of BRCA1/2 mutation carriers of breast cancer patients from WSI and TCGA. *

Category	WSI	TCGA	*P* value
Age [median (IQR)]	54 (45-63)	58 (49-68)	8.6×10^{-11}
Menopause status			
Pre-menopausal	92 (30.5%)	192 (24.2%)	0.038
Post-menopausal	210 (69.5%)	600 (75.8%)	
Grade			
I	40 (8.3%)	51 (8.7%)	0.076
II	165 (34.2%)	238 (40.6%)	
III	277 (57.5%)	297 (50.7%)	
ER			
Positive	366 (65.4%)	677 (76.8%)	3.2×10^{-6}
Negative	194 (34.6%)	205 (23.2%)	
PR			
Positive	313 (56.8%)	588 (66.8%)	1.6×10^{-4}
Negative	238 (43.2%)	292 (33.2%)	
HER2			
Positive	73 (13.0%)	78 (15.3%)	0.30
Negative	487 (87.0%)	431 (84.7%)	
TNBC			
No	388 (70.4%)	422 (83.2%)	1.0×10^{-6}
Yes	163 (29.6%)	85 (16.8%)	
BRCA1/2 status			
BRCA-proficient	483 (86.3%)	874 (94.1%)	2.2×10^{-6}
BRCA1-deficient	47 (8.4%)	31 (3.3%)	
BRCA2-deficient	30 (5.4%)	24 (2.6%)	
BRCA1/2 germline status			
BRCA-proficient	471 (89.5%)	867 (96.4%)	1.2×10^{-6}
BRCA1-deficient	30 (5.7%)	15 (1.7%)	
BRCA2-deficient	25 (4.8%)	17 (1.9%)	
BRCA1/2 somatic status			
BRCA-proficient	471 (95.5%)	867 (97.4%)	0.15
BRCA1-deficient	17 (3.4%)	16 (1.8%)	
BRCA2-deficient	5 (1.0%)	7 (0.8%)	

* Unless otherwise specified, data are presented in no. (%). For each data type, the total number of subjects may differ because of missing or incomplete data. Samples with either genomic or transcriptomic data available included.
