# Supplementary material for: Association of BRCA1- and BRCA2-deficiency with mutation burden, expression of PD-L1/PD-1, immune infiltrates, and T cell-inflamed signature in breast cancer
Source: PLoS One. 2019 Apr 25;14(4):e0215381. doi: 10.1371/journal.pone.0215381 (PMC6483182; doi:10.1371/journal.pone.0215381)
Supplement: S2 Table — (PDF) [file pone.0215381.s006.pdf]

Supplementary table 2: Clinicopathological characteristics and PAM50 subtypes of BRCA1/2-deficient vs. BRCA-proficient breast cancers in WSI and TCGA. *

Category	WSI					TCGA				
	BRCA-proficient	BRCA1-deficient	BRCA2-deficient	*P* value [a]	*P* value [b]	BRCA-proficient	BRCA1-deficient	BRCA2-deficient	*P* value [a]	*P* value [b]
Age [median (IQR)]	56 (47-66)	47 (36-59)	51 (49-61)	4.5×10^{-3}	0.44	59 (49-68)	52 (42-59)	58 (44-65)	1.1×10^{-3}	0.33
Grade										
I	22 (10%)	0	0	5.3×10^{-4}	0.53	50 (9.3%)	0	0	3.6×10^{-3}	0.57
II	88 (41%)	3 (12%)	6 (40%)			226 (42%)	5 (19%)	7 (41%)		
III	106 (49%)	23 (89%)	9 (60%)			264 (49%)	22 (82%)	59 (59%)		
TNBC										
No	174 (78%)	5 (19%)	11 (79%)	1.3×10^{-9}	>0.99	403 (85%)	6 (38%)	13 (81%)	3.2×10^{-5}	0.72
Yes	48 (22%)	22 (82%)	3 (21%)			71 (15%)	10 (63%)	3 (19%)		
PAM50										
Luminal A	94 (42%)	2 (7.4%)	3 (20%)	5.0×10^{-11}	0.33	283 (33%)	0	3 (13%)	6.5×10^{-10}	0.032
Luminal B	80 (36%)	2 (7.4%)	8 (53%)			290 (33%)	7 (23%)	14 (58%)		
Basal-like	38 (17%)	23 (85%)	3 (20%)			142 (16%)	21 (68%)	3 (13%)		
HER2-enriched	11 (4.9%)	0	1 (6.7%)			103 (12%)	2 (6.5%)	1 (4.2%)		
Normal-like	1 (0.4%)	0	0			54 (6.2%)	1 (3.2%)	3 (12.5%)		

* Unless otherwise specified, data are presented in no. (%). For each data type, the total number of subjects may differ because of missing or incomplete data. Only samples with transcriptomic data included.

[a] BRCA1-deficient vs. BRCA-proficient breast cancers

[b] BRCA2-deficient vs. BRCA-proficient breast cancers
