# Supplementary material for: Association of BRCA1- and BRCA2-deficiency with mutation burden, expression of PD-L1/PD-1, immune infiltrates, and T cell-inflamed signature in breast cancer
Source: PLoS One. 2019 Apr 25;14(4):e0215381. doi: 10.1371/journal.pone.0215381 (PMC6483182; doi:10.1371/journal.pone.0215381)
Supplement: S3 Table — (PDF) [file pone.0215381.s007.pdf]

Supplementary table 3: Multiple linear regression of BRCA1/2 status and T-cell inflamed signature score adjusted for clinicopathological features and PAM50 subtypes.

Characteristic	WSI		TCGA	
	β (S.E.)	*P* value	β (S.E.)	*P* value
BRCA1-deficient [a]	0.52 (0.19)	6.1×10^{-3}	-0.03 (0.37)	0.94
BRCA2-deficient [a]	0.33 (0.22)	0.13	-0.28 (0.38)	0.46
BRCA1-deficient [b]	0.32 (0.19)	0.096	0.08 (0.28)	0.77
BRCA2-deficient [b]	0.31 (0.21)	0.14	-0.27 (0.35)	0.44

[a] Adjusted for age, grade, TNBC

[b] Adjusted for age, grade, PAM50 subtypes
